# Optimization of the Detection Method for Phosphorylated α-Synuclein in Parkinson Disease by Skin Biopsy

**DOI:** 10.3389/fneur.2020.569446

**Published:** 2020-09-30

**Authors:** Xiaojing Liu, Jing Yang, Yanpeng Yuan, Qian He, Yuan Gao, Chenyang Jiang, Lanjun Li, Yuming Xu

**Affiliations:** ^1^Department of Neurology, the First Affiliated Hospital of Zhengzhou University, Zhengzhou, China; ^2^Key Laboratory of Cerebrovascular Disease of Henan Province, Zhengzhou, China

**Keywords:** skin biopsy, Parkinson's disease, phosphorylated a-synuclein, section thickness, biopsy sites

## Abstract

**Background:** Recent studies have found deposition of phosphorylated α-synuclein (p-syn) in Parkinson disease (PD) patients' skin, indicating p-syn may be a potential biomarker of PD. However, the sensitivity of the p-syn detection varied largely from 5. 3 to 100%, this influenced the clinical use of this detection method to some extent.

**Objective:** This study aimed to optimize the skin biopsy method for detecting p-syn deposition in patients with PD.

**Methods:** Ninety PD patients and 30 healthy controls underwent skin biopsies at 2–3 of the following sites: the distal leg, thigh, cervical region, or forearm. Skin biopsy samples were cut to 50- and 15-μm thickness sections. Deposition of p-syn were detected by using double immunofluorescence labeling of protein gene production 9.5 (PGP9.5) /p-syn. Statistical data analysis was performed using SPSS 25.0 software.

**Results:** Deposition of p-syn were found in 75/90 PD patients but not in healthy controls (*p* < 0.001). The positive deposition rate of p-syn in the single cervical site was significantly higher than that in the distal leg, thigh, and forearm site. Two samples from the cervical region had a higher p-syn positive rate compared to single cervical site (90.5 vs. 66.7%, *p* = 0.037). There was no significant difference between the p-syn positive rate of samples from the distal leg/cervical sites and 2 samples from cervical region (80 vs. 90.5%, *p* = 0.261). Next, the p-syn positive deposition rate of 2-biopsy samples including distal leg/cervical sites and double samples in the cervical site were comparable to the 3-biopsy samples. The 50-μm section had a significantly higher p-syn positive rate than the 15-μm section (*p* = 0.049).

**Conclusions:** Two biopsy sites (cervical/distal leg) or 2 samples from the cervical site were considered to be priority biopsy sites for detecting p-syn in PD patients. Thick sections may provide a higher p-syn positive rate than thin sections for skin biopsies. These findings provide an optimized p-syn detection method, indicate the valuable pathology biomarker of PD and will promote the clinical use of skin biopsy in the future.

## Introduction

Parkinson disease (PD) is one of the most common neurodegenerative movement disorders (1). Worldwide, its prevalence is ~1% of the population over 60 years old (2), and in China its prevalence is ~1.7% of the population over 65 years old (3). Due to its high prevalence and associated disability rate, PD has been associated with a socioeconomic burden on families and society (4). Therefore, early diagnosis is crucially important.

The main pathology characteristics of PD was the degeneration of dopaminergic neurons and the deposition of Lewy bodies in the substantia nigra. The main component of Lewy bodies is α-synuclein. Studies have reported that phosphorylated α-synuclein (p-syn) was identified by skin biopsy. The deposition morphology characteristics was identical to that of p-syn in the substantia nigra by autopsy (5, 6). P-syn was found in synucleinopathies, such as PD, Lewy bodies (DLB), and multiple system atrophy (MSA), but not in normal controls and taupathies (7–12). These evidences indicated cutaneous p-syn could be a reliable peripheral biomarker for PD diagnosis.

However, we found that the positive rate of p-syn deposits in the skin of PD patients varies from 5.3 to 100% due to the use of different biopsy sites, number of samples, and thickness of sections (5, 9, 13), which affects the diagnostic efficiency in PD, and the clinical application of skin biopsy.

Our study aims to optimize the method for detecting p-syn in the skin of PD patients by comparing the p-syn positive rate of different biopsy sites, the number of samples biopsied, and the thickness of sections.

## Materials and Methods

### Subjects

PD patients irrespective of disease stage were continuously recruited from the in- or outpatient Department of Neurology in the First Affiliated Hospital of Zhengzhou University from February 2019 to February 2020 from skin biopsy database. Diagnosis of PD was based on the 2015 clinically established diagnostic criteria of the Movement Disorders Society (14) and was performed by two professional neurological physicians in accordance. Familial PD patients were not included.

We collected general demographic information of PD patients. The motor symptoms of PD were assessed using the MDS Unified Parkinson's Disease Rating Scale III (MDS-UPDRS-III) (15). Hoehn & Yahr (H-Y) stages were used to define the severity of the disease (16). Thirty healthy participants without any parkinsonian symptoms were recruited as controls. The study was approved by the Ethics Review Committee of the First Affiliated Hospital of Zhengzhou University. All participants provided written informed consent.

### Skin Biopsy

Three-millimeter punch biopsies were used to obtain the skin sites (17, 18). The skin sites included the cervical area (2 cm from the C7 vertebrae), the thigh (15 cm below the trochanter), the distal leg (10 cm above the lateral malleolus), and the forearm (volar). A second cervical skin biopsy was taken adjacent to the first sample in 21 of 90 PD patients. After applying 2% lidocaine local anesthesia using sterile technique, the biopsy samples were fixed in Zamboni's fixation solutions for 18–24 h, and then stored overnight at 4°C overnight in a cryoprotectant solution (19). Frozen sections 50 and 15 μm thick were cut using a Leica cryostat (CM1950, Germany), and every sixth section was chosen for immunostaining; at least four sections were chosen for each specimen. Double immunofluorescence staining was performed by labeling with protein gene product 9.5 (PGP9.5) (1:2,000, mouse, Bio-Rad, USA) and p-syn (1:500, rabbit, Abcam, USA) (20). As secondary antibodies, an anti-mouse Alexa Fluor 594 (1:200, OriGene, USA) and an anti-rabbit Alexa Fluor 488 (1:400, OriGene, USA) were used for detection.

All sections were analyzed and photographed under the Leica fluorescent microscope (DM6000B, Germany). The microscope settings were kept the same for all analyses. The deposition of p-syn was observed under high objective magnification (40 ×). The analysis was performed blindly by two examiners with expertise in immunofluorescence analysis, and the positive criteria reached complete agreement between the two analysts. The p-syn positive deposition ratio was expressed as a percentage of patients who had p-syn deposition in the skin nerves.

### Statistical Analysis

Statistical data analysis was performed using SPSS 25.0 software (IBM, Ehningen, Germany). Categorical data were tested by the χ2 test or Fisher's exact test. Numerical data were expressed as mean ± standard deviation. Non-parametric Mann–Whitney U tests were used to compute the continuous variables. A value of *p* < 0.05 was used to define statistical significance.

## Results

### Demographic Data and Clinical Characteristics

A total of 90 PD patients were included in this study, with a mean age of 59.81 ± 9.15 years (range, 32–87 years). PD patients covered all H-Y stages with an average H-Y stage of 2.07 ± 0.81. Thirty healthy control subjects participated in the study. The demographic data are presented in Table 1.

**Table 1:** Demographic data and clinical characteristics of PD patients.

	**PD (*n* = 90)**	**controls (*n* = 30)**	***p-*value**
Sex	Men = 43, women = 47	Men = 11, women = 19	0.289
Age (years)	59.81 ± 9.15 (32–87)	59.10 ± 10.93 (41–78)	0.726
Age on set (years)	55.88 ± 9.52 (24–82)	–	–
Duration (years)	3.98 ± 3.12 (0.33–15)	–	–
UPDRS-III (scores)	33.46 ± 20.07 (7–95)	–	–
H–Y (stage)	2.01 ± 0.81 (1–5)	–	–

*PD, Parkinson disease; UPDRS, Unified Parkinson's Disease Rating Scale; H–Y, Hoehn and Yahr stage*.

All PD patients received skin biopsies from the leg and cervical region. Among them, 24, 19, and 21 PD patients received an extra biopsy sample from thigh, forearm, and cervical area (as a second cervical sample), respectively. There were no adverse events during or after the skin biopsies. There were no statistical correlations of the UPDRS score (*p* = 0.653), H-Y stage (*p* = 0.932), or the duration of the disease (*p* = 0.877) in PD patients between those with and without p-syn deposits.

### P-syn Positive Deposition

**Figure 1:**
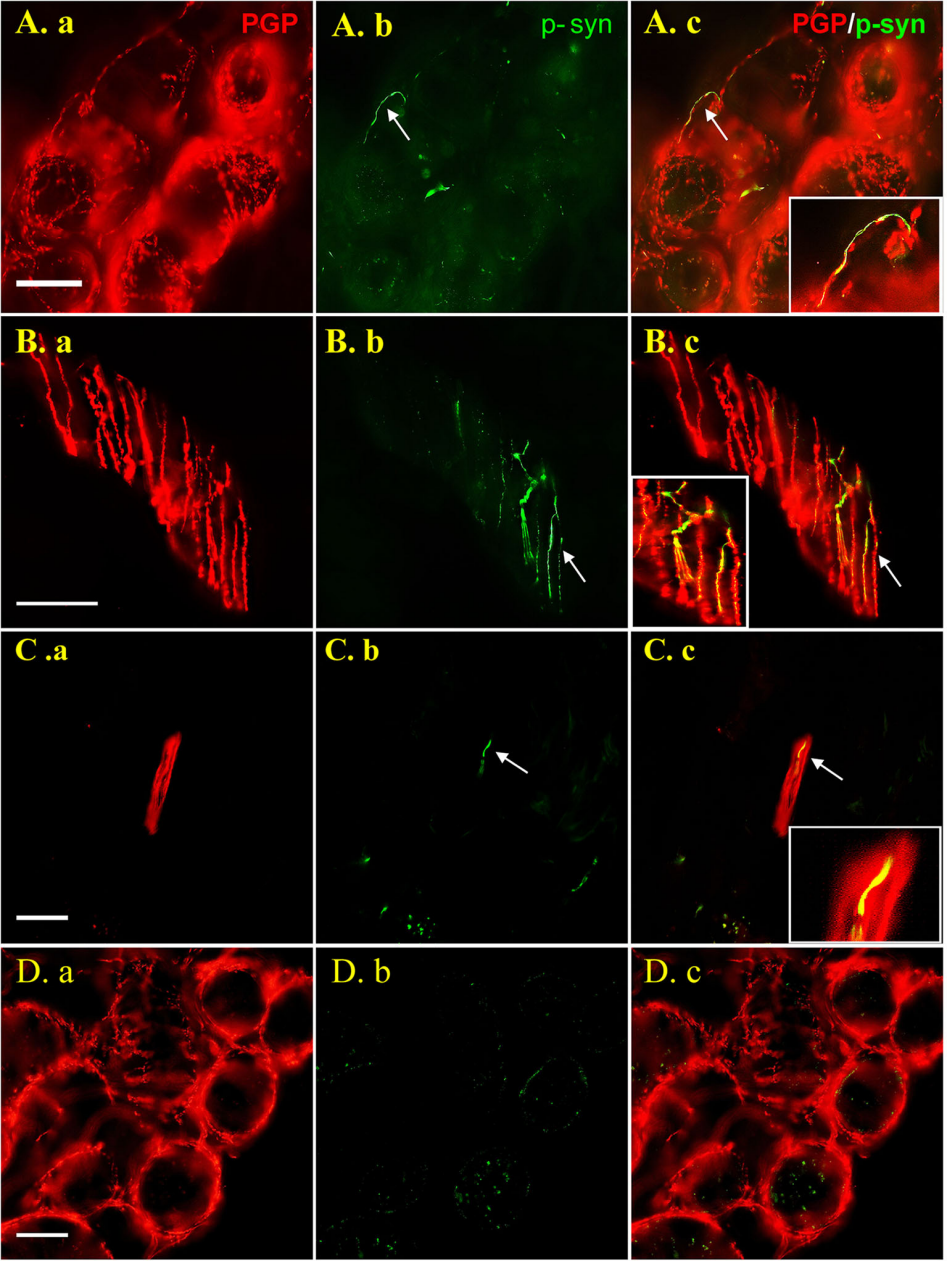
Images are shown for PD patients **(A–C)** and healthy control subjects **(D)**. Double labeling with anti-p-syn (green) and anti-PGP9.5 (red) were shown in 50 μm thick sections **(A,B)** and 15 μm sections **(C)**. P-syn deposits (arrows) can be seen in nerve fibers innervating sweat gland (A.b), arrector pili muscle (B.b) and dermal nerve bundle (C.b) labeling by PGP9.5 (A.a, B.a, C.a). No p-syn deposition (D.b) was observed in sweat gland of the control (D.a). Figures were merged and magnified in A.c, B.c, C.c, and D.c. Bar = 50 μm. White arrow indicate p-syn positive deposition or PGP9.5/p-syn double positive position. PGP9.5: protein gene product 9.5; p-syn: phosphorylated alpha synuclein.

Dot-like or linear p-syn immunosignals colocalized with PGP9.5 signals in 75/90 (83.3%) PD patients, but in none of the controls (*p* < 0.001). P-syn deposits were found in nerve bundles in both superficial and deep dermis, subepidermal plexus, and nerve fibers innervating blood vessels, sweat glands, arrector pili muscles and hair follicles, but not in intraepidermal nerve fibers. P-syn deposition in nerve fibers innervating sweat glands (A.b) and arrector pili muscles (B.b), and nerve bundles in deep dermis (C.b) was shown as representation in Figure 1.

#### Single-Biopsy Site

The cervical site had a higher p-syn positive rate than the leg (42.2%, 38/90, *p* = 0.001), the thigh (33.3%, 8/24, *p* = 0.004), and the forearm site (21.1%, 4/19, *p* < 0.001) among single-biopsy sites.

#### Two Biopsy Sites or Samples

The p-syn positive rate of the leg/forearm sites was significantly higher than that of the forearm site (*p* = 0.044), and it was comparable with the cervical (single sample) or thigh site. Leg/cervical sites showed the highest p-syn positive rate in 2-site biopsies (1 sample in each site), which had significantly higher p-syn positive rate of the single leg (*p* = 0.001), thigh (*p* = 0.004), and the forearm (*p* = 0.003), respectively.

The 2-site combined cervical/leg biopsy (80%) had significantly higher p-syn deposition than thigh/leg (*p* = 0.001), forearm/leg (*p* = 0.012), cervical/forearm (*p* < 0.001), and cervical/thigh (*p* = 0.021). Two samples taken from adjacent areas of the cervical site had higher p-syn positive rate compared with a single sample from this biopsy site (90.5 vs. 66.7%, *p* = 0.037). The p-syn positive rate was not different between 2-site leg/cervical biopsies and double samples from the cervical site (*p* = 0.261, Table 2).

**Table 2:** The p-syn positive ratio in one and biopsy sites.

	**Single site**	**Two sites (increase cervical site)**	***p-*value**	**Two sites (increase forearm)**	***p*-value**	**Two sites (increase thigh)**	***p-*value**	**Two sites (increase leg)**	***p-*value**
Positive ratio	Cervical 66.7% (61/90)	Cervical x 290.5% (19/21)	0.037^*^	Cervical/forearm68.4% (13/19)	0.956^*^	Cervical/thigh75% (18/24)	0.496	Cervical/leg80% (72/90)	0.062
	Leg 42.2% (38/90)	Leg/cervical80% (72/90)	<0.001^*^	Leg/forearm52.6% (10/19)	0.406	Leg/thigh45.8% (11/24)	0.751	–	
	Thigh 33.3 (8/24)	Thigh/cervical75% (18/24)	0.004^*^	–	–	–	–	Thigh/leg45.8% (11/24)	0.376
	Forearm 21.1% (4/19)	Forearm/cervical68.4% (13/19)	0.003^*^	–	–	–	–	Forearm/leg52.6% (10/19)	0.044^*^
*p-*value	<0.001^*^	0.521		0.265		0.039^*^		0.001^*^	

* *p < 0.05*.

#### Three Biopsy Sites or Samples

The 3-site biopsies with the addition of the cervical region to the leg/forearm (84.2 vs. 80%, *p* = 0.672) or thigh/leg (75 vs. 80%, *p* = 0.593) had higher positive rates than the 2-site biopsies. Combination of a third biopsy site of the forearm (84.2 vs. 52.6%, *p* = 0.036) or thigh (75 vs. 45.8%, *p* = 0.039) did not increase the p-syn positivity of the 2-site cervical/leg biopsy. Three samples, including two from the cervical region and one from the leg, did not further increase the p-syn positivity of the double samples from the cervical region (95.2 vs. 90.5%, *p* = 0.549) or 2-biopsy cervical/leg sites (95.2 vs. 80%, *p* = 0.095; Table 3).

**Table 3:** The p-syn positive ratio between two and three biopsy site(s).

**Positive ratio**								
**Three** **sites**	**Distal leg/cervical/forearm** **84.2% (16/19)**			**Distal leg/cervical/proximal thigh** **75% (18/24)**			**Cervical** **×** **2/distal leg** **95.2% (20/21)**	
Two sites	Distal leg/cervical 80% (72/90)	Cervical/forearm68.4% (13/19)	Distal leg/forearm 52.6% (10/19)	Distal leg/cervical80% (72/90)	Cervical/proximal 75% (18/24)	Distal leg/proximal45.8% (11/24)	Distal leg/cervical 80% (72/90)	Cervical **×** 290.5% (19/21)
*p-*value	0.672	0.252	0.036^*^	0.593	1.000	0.039^*^	0.095	0.549

* *p < 0.05*.

#### Thickness of Specimen

Thirty-two specimens (16 from the cervical site, 16 from the leg site) from 16 PD patients were used to detect the p-syn positive deposition rate using section slices of 15- or 50-μm thickness. The 50-μm thick section had a higher p-syn positive rate than the 15 μm section in cervical biopsies (*p* = 0.049), but no differences were found in the leg or leg/cervical sites (Table 4).

**Table 4:** The p-syn deposition in different section thickness.

	**50 μm positive ratio (%)**	**15 μm positive ratio (%)**	***p-*value**
Leg	56.3% (9/16)	31.3% (5/16)	0.154
Cervical	87.5% (14/16)	56.3% (9/16)	0.049
Leg + cervical	87.5% (14/16)	81.3% (13/16)	0.626

## Discussion

Our study systematically compared the p-syn positive rate of different skin biopsy sites, number of biopsy samples, and different thicknesses of sections in PD patients. The main results were as follows: (1) the highest p-syn positive rate was found in the cervical single-biopsy site; (2) 2-site leg/cervical biopsies and double samples from the cervical site had comparable p-syn positive rates, which were significantly higher than other 2-sample combinations in this study; (3) combination of a third biopsy site using the forearm or thigh did not increase the p-syn positivity of the 2-site cervical/leg biopsy; (4) the 50-μm thick sections had a higher p-syn positive rate than the 15 μm sections.

Skin biopsies are minimally invasive, easy to perform, and allow multiple samples to be taken (17, 21). Using these advantages, we compared the p-syn positive deposition rate of PD patients via biopsy at different sites, number of samples, and in sections with different thicknesses. Previous studies presented p-syn deposition in PD patients as a patchy deposition (22), indicating that multiple biopsy sites or samples could increase the detection of p-syn positive rate. However, no data were available about how many biopsy sites or samples were enough to satisfy both higher p-syn sensitivity and the mini trauma. The biopsy sites in our study were chosen based on those reported by former studies that included hairy and hairless areas.

The results showed that the cervical site has the higher positive rate of p-syn than the thigh and distal leg which is consistent with the results of Donadio et al. and Mili et al. (9, 10). It was more likely to deposit in the proximal ganglion. The study of Melli et al. suggested that the cervical region has more nerve fibers (10) due to the length-dependent distribution (5).

Because small fiber neuropathy often occurred in PD patients, the leg was the recommended biopsy site for counting intraepidermal nerve fiber density and was considered the routine biopsy site (18). In our study, the p-syn positive rate of 2-site leg/cervical biopsy was significantly higher than the leg single site, and the double samples from the cervical site had a higher p-syn positive rate than 1-site biopsy, in accordance with Donadio et al. (9), due to the patchy-like deposition. The p-syn positive rate of the leg/cervical two sites and the double samples in cervical were comparable, and p-syn detection could not be increased further when three biopsy samples were taken. Therefore, we would recommend that two biopsy sites (cervical/leg) or two samples from the cervical site be considered priority biopsy regions for detecting the p-syn in PD patients.

Zange et al. (8) found that the positive rate of p-syn in the skin of the forearm, which was supposed to the hairless area, could reach to 100% in PD patients. However, our data only showed 21.4% positive rate of p-syn, which was far lower than that of Zange et al., indicating that the hairless area may be not a suitable biopsy site for detecting of p-syn, which is in accordance with Doppler et al. One possible reason for this may be that Zange et al. used the paraffin-embedded immunohistochemical staining method and we used the free frozen immunofluorescence method. However, further study is needed to confirm this hypothesis.

The thickness of most commonly used frozen sections were compared (5, 7, 10, 22, 23). In our earlier study, 20-μm thick sections consistent with the study by Doppler et al. were used; however, they fell off the slide too easily. Therefore, in this study, 15-μm thick sections were finally chosen for comparison with the 50-μm thick sections. The results show that thicker sections had a higher p-syn positive rate than thinner sections in PD patients. In addition, there was a tendency that p-syn was more likely to be detected in both the cervical and distal leg sites using 50-μm sections, but in only one of the sites using 15-μm thick sections. We are of the opinion that this may be related to 50-μm sections containing more nerve fibers than 15-μm sections, although thick sections often showed lower discrimination from the background and weaker staining, probably because the difficulty of the antibodies to penetrate into the tissue, than thin sections (24).

Our study also existed some limitations. The participants recruited were all diagnosed according to clinical criteria without pathological confirmation by postmortem. However, this study provides systematic data on the skin biopsy method for detecting p-syn that supports the valuable potential of skin biopsy in early PD diagnosis, which may promote the clinical use of skin biopsies in the future.

## Data Availability Statement

All datasets presented in this study are included in the article/supplementary material.

## Ethics Statement

The studies involving human participants were reviewed and approved by Ethics Committee of the First Affiliated Hospital of Zhengzhou University. The patients/participants provided their written informed consent to participate in this study.

## Author Contributions

YX and JY contributed to conception, design of the study and manuscript revision. YX, JY, and YG contributed to supplement of the funding. XL, YY, QH, CJ, and LL organized the database, did the biopsies. XL, YY, and QH performed the experiments. XL, JY, YY, and YG performed the statistical analysis. XL wrote the manuscript. All authors contributed to the article and approved the submitted version.

## Conflict of Interest

The authors declare that the research was conducted in the absence of any commercial or financial relationships that could be construed as a potential conflict of interest.
